# Neural Activity during Voluntary Movements in Each Body Representation of the Intracortical Microstimulation-Derived Map in the Macaque Motor Cortex

**DOI:** 10.1371/journal.pone.0160720

**Published:** 2016-08-05

**Authors:** Noriyuki Higo, Nobuo Kunori, Yumi Murata

**Affiliations:** 1 Human Informatics Research Institute, National Institute of Advanced Industrial Science and Technology (AIST), Tsukuba, Ibaraki, 305–8568, Japan; 2 Precursory Research for Embryonic Science and Technology (PRESTO), JST, Kawaguchi, Saitama, 332–0012, Japan; 3 Graduate School of Comprehensive Human Science, University of Tsukuba, Tsukuba, Ibaraki, 305–8574, Japan; The University of Melbourne, AUSTRALIA

## Abstract

In order to accurately interpret experimental data using the topographic body map identified by conventional intracortical microstimulation (ICMS), it is important to know how neurons in each division of the map respond during voluntary movements. Here we systematically investigated neuronal responses in each body representation of the ICMS map during a reach-grasp-retrieval task that involves the movements of multiple body parts. The topographic body map in the primary motor cortex (M1) generally corresponds to functional divisions of voluntary movements; neurons at the recording sites in each body representation with movement thresholds of 10 μA or less were differentially activated during the task, and the timing of responses was consistent with the movements of the body part represented. Moreover, neurons in the digit representation responded differently for the different types of grasping. In addition, the present study showed that neural activity depends on the ICMS current threshold required to elicit body movements and the location of the recording on the cortical surface. In the ventral premotor cortex (PMv), no correlation was found between the response properties of neurons and the body representation in the ICMS map. Neural responses specific to forelimb movements were often observed in the rostral part of PMv, including the lateral bank of the lower arcuate limb, in which ICMS up to 100 μA evoked no detectable movement. These results indicate that the physiological significance of the ICMS-derived maps is different between, and even within, areas M1 and PMv.

## Introduction

It is generally accepted that the motor cortex has a topographically organized map of body parts, which is often identified by repetitive intracortical microstimulation (ICMS). During ICMS of the motor cortex, application of a small electrical current evokes involuntary twitches of specific body parts. The topographic body map derived by ICMS has long been thought to reflect the somatotopic organization of motor output from the motor cortex [1–4] and is used to determine the locations for placing anatomical tracers [5–11] as well as for inducing lesions [12–20]. Moreover, alterations in the ICMS map are observed during the course of motor learning and rehabilitative training [15–21], suggesting that they partly reflect the neural changes that underlie motor memory and functional recovery.

Although the ICMS map has been exploited in various fields of neuroscience, the neurophysiological and anatomical underpinnings of the map, *i*.*e*., the neural circuits involved in each body representation of the map and the nature of their involvement in voluntary movements, remain unclear. For example, ICMS may activate not only output projections but also other neuronal elements including axonal collaterals and terminals [22, 23]. In addition, simultaneous recording analyses have shown that both excitatory and inhibitory effects are induced in neighboring neurons and neurons in other cortical areas after ICMS [24–27]. Moreover, ICMS can produce excitation at sites distant to the stimulation site by trans-synaptic excitation that results from temporal summation within the cortical circuitry [27, 28]. Finally, there is debate about whether the motor cortex contains a body map used to control movements of each body part at all [29, 30]. ICMS has conventionally consisted of 10–13 cathodal or biphasic pulses delivered at 330–350 Hz (0.2 ms per phase). However, stimulation with different parameters evokes movements different from those observed with conventional ICMS; for example, high-frequency stimulus trains (200 Hz) lasting 500 ms produce hand movements to consistent endpoint positions around the monkey’s workspace [31–33]. The ICMS map obtained by using this high-frequency and long-duration electrical stimulation may reflect functional divisions distinct from those indicated by the conventional ICMS map, and has also been used to determine where to place anatomical tracers [34].

A proper understanding of the cumulative experimental data acquired with conventional ICMS requires knowledge of how neuronal firing rates in each division of the ICMS map change during voluntary movements that involve multiple body parts. A previous study investigated the somatotopic organization in area F2 of the macaque dorsal premotor cortex (PMd) not only by using ICMS but also based on neuronal responses observed by single-unit recordings during voluntary movements (Raos et al. 2003). The study showed that the somatotopic organization of voluntary movements was similar to that obtained by ICMS, whereas neurons in the most medial sector, where ICMS evoked no movement, were activated during arm and trunk movements. Another study investigated neuronal responses in PMd and the ventral premotor cortex (PMv) during a reach-to-grasp task. In this study, the response latencies did not differ between recording sites where ICMS evoked movements of the proximal forelimb joints and those where it evoked movements of the distal joints, even though the proximal joints moved before the distal joints during the task [35]. These studies provided important information for understanding functional divisions within the premotor cortices. In the present study, we performed single-unit recordings in both the primary motor cortex (M1) and PMv during a reach-grasp-retrieval task which involves movements of the mouth as well as both the proximal and distal joints of the forelimb, and investigated how neurons in each body representation of the ICMS map differentially responded in the course of the voluntary movement task. The present study showed that the ICMS map in M1 generally corresponds to the functional divisions of voluntary movements, and that neural activity depends on the ICMS current threshold required to elicit body movements. In PMv, no correlation was found between the body representation in the ICMS map and the response properties of neurons.

## Materials and Methods

### Subjects

Two adult Japanese monkeys (*Macaca fuscata*), weighing 7 kg (Monkey B, female) and 10 kg (Monkey G, male), were used in the present study. The monkeys were purchased from a local provider (Kawahara Bird-Animal Trading Co. Ltd., Tokyo, Japan). No statistical methods were used to pre-determine sample sizes. We attempted to minimize the number of monkeys used on the basis of ethical considerations and data similarity; our sample sizes are similar to those reported in previous publications by our group and others. Naive monkeys without any history of experimentation were used. The protocol of the present study was approved by the Institutional Animal Care and Use Committee of the National Institute of Advanced Industrial Science and Technology (AIST), Japan (#32-08-004, #2010-049A), conformed to the NIH Guidelines for the Care and Use of Laboratory Animals and ARRIVE guidelines (S1 ARRIVE Guidelines Checklist). Our procedure also followed the recommendations of the Weatherall report [36]. The monkeys were housed in adjoining individual primate cages that allowed social interactions under controlled conditions of humidity, temperature, and light; they were monitored daily by the researchers and animal care staff to ensure their health and welfare. Environmental enrichment consisted of commercial toys. A commercial primate diet and fresh fruit and vegetables were provided daily, and water was provided in a drinking bottle and freshened daily. No monkey was killed for this study. Both monkeys will be used as subjects for another study. All surgery was performed under sodium pentobarbital anesthesia, and all efforts were made to minimize suffering.

### Behavioral task

**Fig 1:**
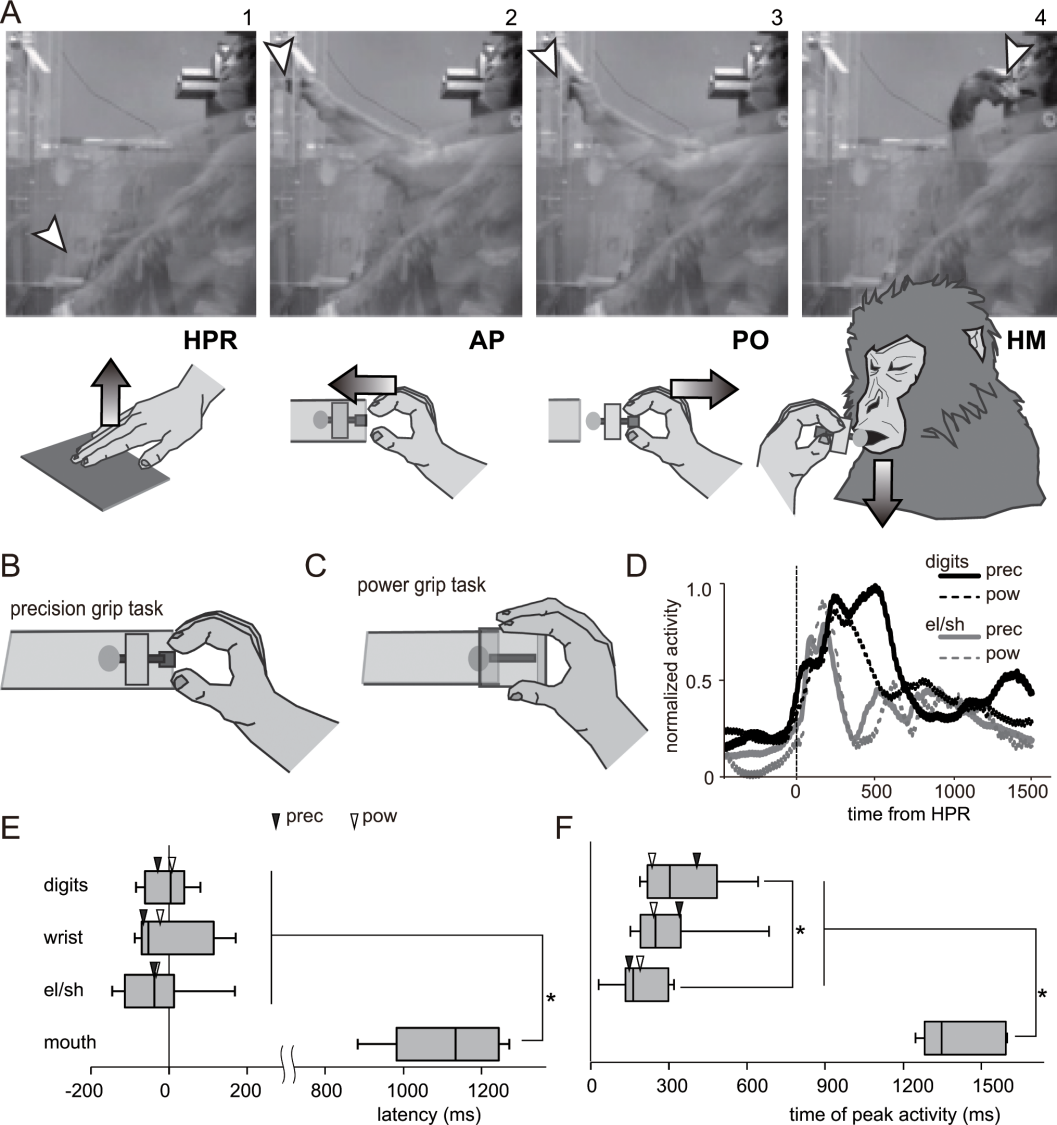
Experimental setup and the hand movements of the monkeys. (A) Sequence of photographs and drawings during a single trial of the reach-grasp-retrieval task. Panels 1, 2, 3, and 4 show the moments at which the monkey released a homepad (HPR), reached toward the target (AP), pulled the target out (PO), and brought its hand to its mouth (HM), respectively. The arrowheads in the photographs indicate the locations of the fingertip. The arrows in the drawings indicate the movement direction of the hand at HPR, AP, and PO, and that of the mouth at HM. (B, C) Schematic illustrations showing the precision grip (B) and power grip (C) tasks. In the precision grip task, the monkey retrieved a disk from a vertical slit aperture of the tube by grasping a small knob (7 × 7 × 7 mm in size), using a precision grip with the tips of its index finger and thumb. In the power grip task, the monkey retrieved a cylindrical apparatus (32 mm in diameter and 30 mm in length), using a power grip. (D) Activity of digit (digits: flexor digitorum superficialis) and shoulder muscles (el/sh: biceps brachii) during the precision (prec) and power (pow) grip tasks, aligned to the time of HPR. EMG was normalized to the maximum level of activity across two grasp types. Activity of proximal forelimb muscles increased around the time of HPR, and that of distal forelimb muscles increased during grasping of the object, 300–500 ms after HPR. The proximal forelimb muscles are more active during retrieval of the object, 500–1000 ms after HPR, than during the preceding period of grasping. (E, F) Sequential activation of the muscles of the forelimb and mouth during task performance. The box and whisker plots show the median, upper and lower quartiles, and 10th and 90th percentiles of the latencies (E) as well as the times of peak activity (F) relative to HPR. Both latency and peak activation time of the mouth muscle were significantly greater than those of the forelimb muscles, and the peak activation time of the digit muscle was significantly greater than that of the elbow/shoulder muscles (*P < 0.01, Mann–Whitney U-test). The black and white triangles indicate the median values of latency and time of peak activity for precision and power grip tasks, respectively.

Monkeys were trained on a reach-grasp-retrieval task in which the animal pressed a homepad with the hand, released the hand, and then reached for a target object to which a food morsel was attached. The monkey then grasped and retrieved the food morsel, moved its hand to its mouth, and then ate the morsel (Fig 1A). The experimenter placed the object back into the cylinder at the end of every trial.

We first determined the preferred hand of monkeys by recording the hand that was used for reaching and grasping the target object, as in our previous studies [12, 13]. We defined the preferred hand as that used on >25 of 30 daily trials over 2 consecutive days. The preferred hand for Monkey B was the right and that for Monkey G was the left. The monkey sat in the primate chair with the head fixed to the chair. The non-preferred hand was restrained during task performance. At the start of each trial, the monkey used the preferred hand to press a homepad, which was horizontally installed on the chair at the height of the monkey’s waist. After a variable delay (200–800 ms), the shutter in front of the monkey was opened for 2500 ms. Behind the shutter, one of two types of target to retrieve was located at shoulder height 15 cm in front of the primate chair. In the precision grip task, a disk (20 mm in diameter) with a small knob (7 × 7 × 7 mm in size) attached to the front side and a piece of sweet potato attached to the other (Fig 1B), was positioned in the center of a slit (10 mm in width). The monkey retrieved the disk from the vertical slit aperture of the tube by grasping the knob using a precision grip, with the tips of the index finger and thumb. In the power grip task, a piece of sweet potato was attached to the rear side of a cylindrical apparatus (32 mm in diameter and 30 mm in length) (Fig 1C). The monkey retrieved the apparatus by using a power grip, after which it moved the hand to the mouth and ate the piece of sweet potato attached.

The time of homepad release (HPR) was detected by a touch sensor (HTS-30, Sensatec, Kyoto, Japan). The time of approaching the target (AP) and that of pulling the target out (PO) were detected by a laser fiber sensor (LV-11SB with sensor head LV-S72, Keyence, Osaka, Japan) installed at the aperture of the tube from which the retrieval targets were presented. The onset and duration of reaching movements made toward the target were detected by the trial event markers for HPR and AP, and those of grasping and retrieving by the trial event markers for AP and PO. The time at which the monkey’s hand arrived at the mouth (HM) was detected by another laser fiber sensor (LV-11SB, Keyence) installed just in front of the monkey’s mouth.

### Single-unit recording and ICMS

After the monkeys were trained to perform the reach-grasp-retrieval task, a craniotomy was made over M1 (for Monkey B) or both M1 and PMv (for Monkey G) in the hemisphere contralateral to the monkeys’ preferred hand under sterile conditions and pentobarbital anesthesia (25 mg/kg), as in our previous studies [12, 13]. The locations of M1 and PM were determined using stereotaxic coordinates from magnetic resonance images of each monkey’s brain using a 3.0T MRI system (3T Signa LX; General Electric Medical Systems, Milwaukee, WI). A stainless steel chamber and head holders were then affixed to the skull with dental acrylic. After the operation, the monkeys were treated subcutaneously with meloxicam (0.2 mg/kg) and monitored continuously in a warmed environment until they had fully recovered from the anesthesia. No adverse events were seen in the present study. First, the ICMS maps of M1 and PMv were roughly constructed to determine the cortical locations where single-unit recording was to be performed. A flexible parylene-insulated tungsten microelectrode (MicroProbe, Carlsbad, CA) was advanced perpendicular to the dura to a depth of 1–10 mm by using a hydraulic microdrive (MO95-S, Narishige, Tokyo, Japan). During electrode penetrations, the first cortical unit activity was noted. At intervals of 1 mm in depth, we recorded body movements evoked by conventional ICMS, using parameters that have frequently been used in previous studies [5–7, 10–13]. The sensory response was also investigated to determine the border between the primary motor and sensory areas, using light tactile stimuli to the face, digit, wrist, and forearm of the monkey with a small hand-held brush as in our previous studies [12, 13].

After the ICMS maps of M1 and PMv were roughly constructed, a flexible parylene-insulated tungsten microelectrode (MicroProbe) was penetrated. The sites of penetration on the cortical surface were spaced 1 mm apart. The microelectrode was advanced perpendicular to the dura, and neural signals from this electrode was amplified, filtered, and displayed by standard methods, using a head amplifier (JH-110J, Nihon Kohden, Tokyo, Japan), main amplifier (MEG-5100, Nihon Kohden), and digital oscilloscope. Spikes were isolated using a time-amplitude window discriminator (Bak Electronics, Mount Airy, MD) and converted to voltage pulses. The data were then sent to a TEMPO data acquisition system (Reflective Computing, St. Louis, MO), which saved spike time stamps to a hard disk along with the trial event markers HPR, AP, PO, and HM. The sampling frequency was 1 kHz for both spikes and trial event markers. Data from each single unit were recorded for 15 trials for each block of the precision and power grip tasks—a total of 30 trials for each single unit. Four to six single units, spaced across more than 400 μm in depth, were recorded for each penetration.

The body representation of each recording site was confirmed just after the single-unit recording by using conventional ICMS (a train of 12 monophasic cathodal pulses, 200 μs in duration at 333 Hz). In M1, body movements evoked by the electrical current at 50, 30, 20, 15, 10, and 5 μA were noted to determine the movement threshold. In PMv, where ICMS at 50 μA evoked no movement, body movements evoked by the electrical current at 100, 80, 70, and 60 μA were noted. Compared with inspection by electromyogram (EMG), our use of visual inspection to characterize the responses to ICMS is likely to overestimate the threshold.

For EMG recordings, a small bipolar surface electrode (Carefusion, Middleton, WI) was placed along the longitudinal axis of the following muscles: temporalis, masseter, trapezius, deltoid, biceps brachii, triceps brachii, brachioradialis, flexor carpi ulnaris, flexor carpi radialis, extensor carpi ulnaris, extensor carpi radialis longus/brevis, flexor digitorum superficialis, flexor hallucis longus, or extensor digitorum communis. EMG signals were recorded using a Personal-EMG electromyograph (Oisaka Electronic Device Ltd, Fukuyama, Japan) while animals performed the reach-grasp-retrieval task. The recorded EMG data were then sent to a TEMPO data acquisition system (Reflective Computing), along with the trial event markers for HPR, AP, PO, and HM. The sampling frequency was 1 kHz for both EMG data and trial event markers. EMG recordings were performed in sessions separate from those in which neuronal activity was recorded. The total number of EMG recordings was 88. During all experiments (single-unit and EMG recordings as well as ICMS), the monkey’s body movements were recorded by six video cameras (one HDC-TM350 camera, Panasonic, Osaka, Japan; five WAT-902H Ultimate cameras, Watec, Tsuruoka, Japan) installed around the task apparatus.

### Data analysis

We recorded 647 neurons in M1 (310 for Monkey B and 337 for Monkey G). Among them, the data for 414 M1 neurons at recording sites where ICMS up to 50 μA evoked movements of the digits, wrist, elbow, shoulder, or mouth were used for most of the analyses. The data in the elbow and shoulder representations were pooled to have a sufficient number of data points. All results in M1 were consistent between Monkeys B and G and are therefore reported together unless otherwise specified. The results from the precision and power grip tasks were also pooled for the EMG analysis because only the time of peak activity in digit muscles differed significantly between them (*P* < 0.01, Mann–Whitney U test); no significant difference in muscle activity between precision and power grip tasks was observed for latency, time of peak activity, maximum activity, or length of time period when the activity significantly changed from baseline. In the analyses using Forelimb Movement Index (FMI, see below), data for M1 neurons in the recording sites where ICMS up to 50 μA evoked no movement were used. Data for neurons in PMv were also analyzed in Monkey G. PMv neurons were defined as neurons at recording sites rostral to M1, caudal to the arcuate sulcus, and ventral to the arcuate spur. We recorded 115 neurons in PMv, and performed ICMS after recording. ICMS up to 100 μA at the recording sites of 5 out of 115 neurons elicited digit or elbow movements. All PMv neurons were used in the analyses.

Data analysis was performed using MATLAB (MathWorks, Natick, MA). First, neural responses were aligned to the time of HPR, and the baseline firing rate was defined as the mean firing rate from 500 to 300 ms before HPR. The baseline activity was not significantly different between precision grip and power grip trials (*P* > 0.3, Mann-Whitney U-test). Neurons were deemed to show task-related activity if the firing deviated significantly from baseline for five consecutive 10-ms time bins (*P* < 0.01, Mann–Whitney U test). The latency and peak firing rate from HPR were calculated, using the periods of task-related activity. EMG data were also aligned to the time of HPR, and the response latency and peak activity from HPR were calculated using the same method as that used for neural activity. Neural data were also aligned to the event markers AP, PO, and HM, and the periods with task-related activity were determined.

To analyze how neuronal activity depends on the movement threshold, the neuronal response properties were compared across recording sites with five different current thresholds for eliciting movements. In this analysis, the percentage of neurons activated and the normalized neuronal activities in the digit representation of M1 during the grasping phase, 0 to +300 ms from AP, were calculated. In addition, to investigate differences in neural activity during precision gripping and during power gripping, the firing rate aligned to AP was normalized by subtracting the baseline firing rate and then dividing the resulting value by the maximum level of activity observed across the two grasp types for all periods.

Finally, we calculated an index that represents how neural activity specifically changes during forelimb movements:

Forelimb Movement Index (FMI) = max [significant peak firing rate (Hz) ±300 ms from HPR, AP, and PO, for both precision and power grip tasks]–baseline firing rate (Hz). / max [significant peak firing rate (Hz) ±300 ms from HPR, AP, and PO, for both precision and power grip tasks] + baseline firing rate (Hz).

FMI translates the neural response into a scale of –1 (strong inhibition) to + 1 (strong excitation). Note that FMI was 0 for neurons that showed no statistically significant change in activity during the 300 ms from HPR, AP, or PO.

### Statistical analyses

Statistical significance was assessed by nonparametric tests including the Mann-Whitney U-test, chi-squared test, and Kruskal–Wallis one-way analysis of variance (ANOVA). Dunn’s test was used for post-hoc analysis of multiple comparisons.

## Results

### Response properties of neurons in each division of the ICMS map in M1

During the course of the reach-grasp-retrieval task (Fig 1A), the monkey released its hand from the homepad (panel 1, HPR); reached for (panel 2, AP), grasped, and retrieved (panel 3, PO) the target object; moved the hand to the mouth (panel 4, HM); and ate the attached food morsel. The movements of wrist and elbow/shoulder preceded those of digits during reaching, around the time of HPR, and the peak activation time was significantly earlier than that of the digit muscles, which are mainly involved in grasping (*P* < 0.01, Mann–Whitney U-test, Fig 1D–1F). Movements of the mouth were observed around the final phase of the task trial, 1000–1500 ms from HPR, and both the latency and peak activation time of the mouth muscles were significantly greater than those of the forelimb muscles (*P* < 0.01, Mann–Whitney U-test, Fig 1E and 1F).

**Fig 2:**
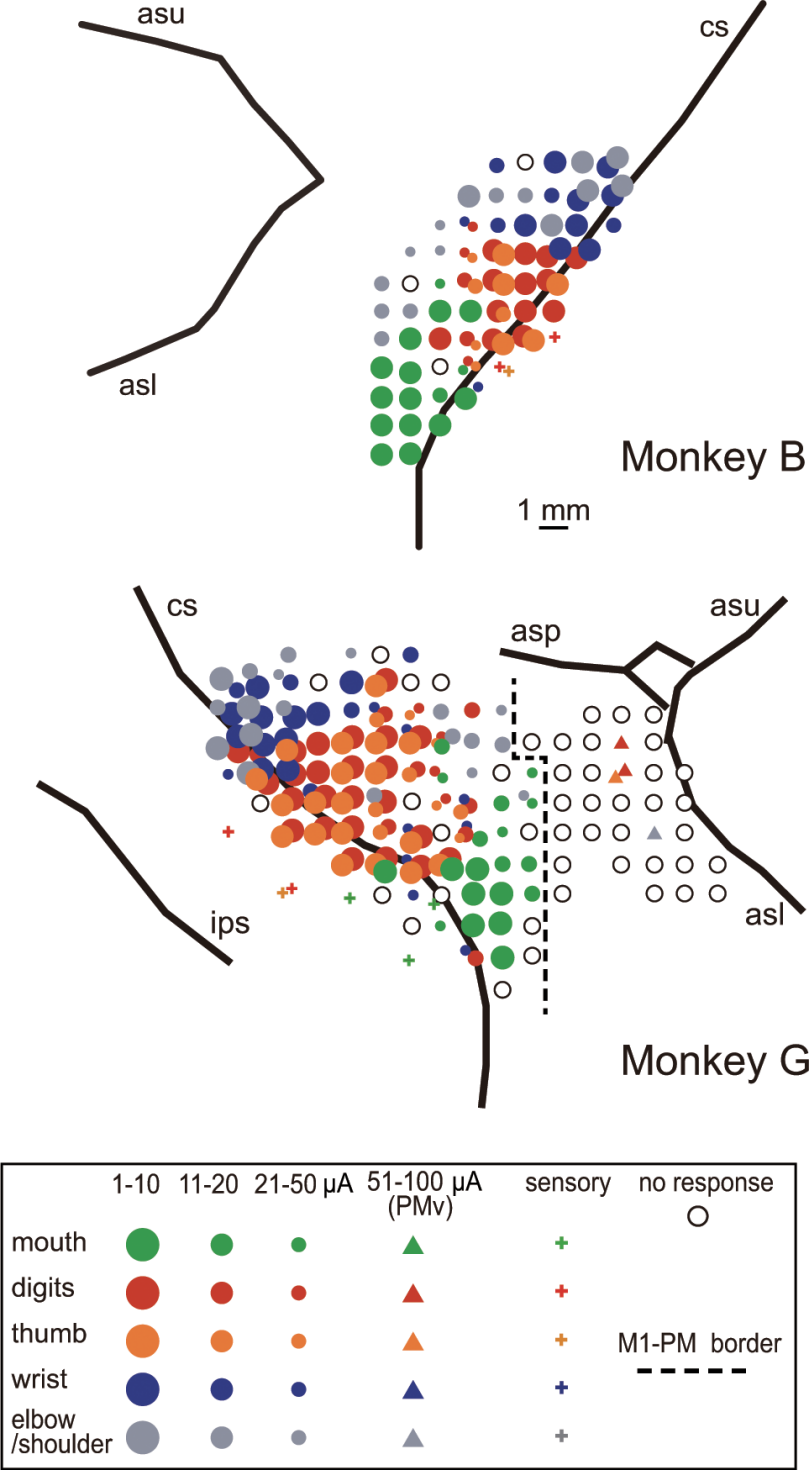
The intracortical microstimulation (ICMS) maps of the monkeys. Movement elicited at the indicated thresholds and the sensory response to light tactile stimuli are indicated by the symbols. Some electrode penetration sites showed no response to either ICMS at currents up to 50 μA for M1 (or up to 100 μA for PMv) or sensory stimulation (open circles). The dashed line indicates the presumed border between M1 and the premotor cortex, which was determined by the movement thresholds and sulcal landmarks. asl, lower limb of the arcuate sulcus; asp, arcuate spur; asu, upper limb of the arcuate sulcus; cs, central sulcus; ips, intraparietal sulcus.

**Fig 3:**
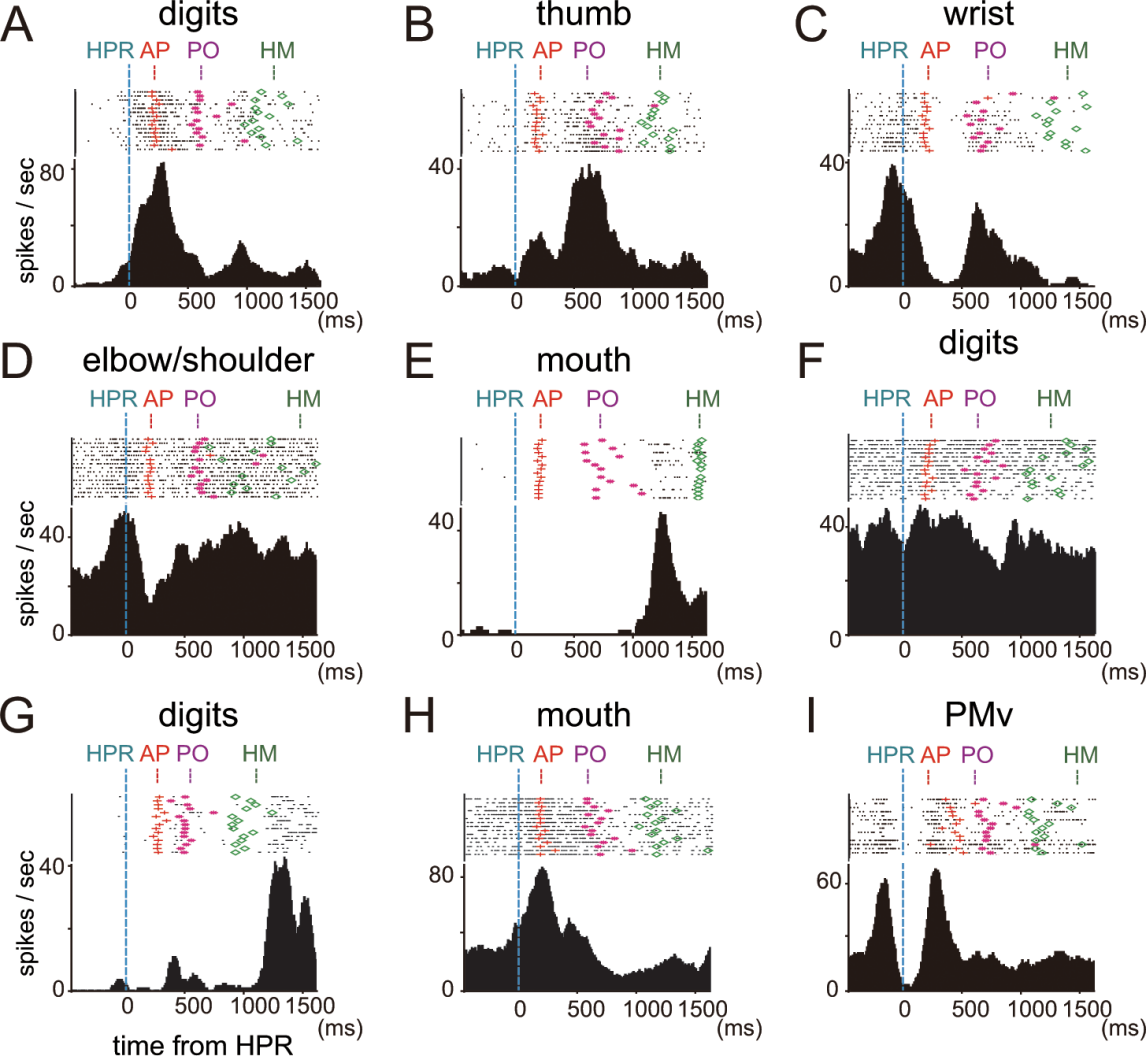
Single-unit recordings in M1 and PMv during the motor task. (A–E) Examples of neurons whose firing rates changed during the task. The timing of responses was consistent with the movements of the body part represented in the recording site. Raster plots and firing rate histograms show examples of neuronal firing during the precision grip task, aligned to the time of HPR (dashed blue lines), in each body representation—digits (A), thumb (B), wrist (C), elbow/shoulder (D), and mouth (E)—of the intracortical microstimulation (ICMS) map in the primary motor cortex (M1). The red crosses, pink asterisks, and green diamonds in the raster plot indicate the times of approaching the target (AP), pulling the target out (PO), and bringing the hand to the mouth (HM), respectively, in each trial. (F–H) Examples of neuronal responses that did not correspond to the body representation on the ICMS map; a neuron in the digit representation that showed no modulation during the task (F), and neurons in the digit and mouth representations showed peak activity in the periods around HM (G) and AP (H), respectively. The higher threshold (HT) neurons shown in (F–H) were located at recording sites with movement thresholds of at least 15μA. (I) A raster plot and firing rate histogram showing examples of neuronal firing during the precision grip task, aligned to the time of HPR, in the ventral premotor cortex (PMv).

We investigated neuronal responses in each body representation of the ICMS map during this reach-grasp-retrieval task. Of the 647 neurons recorded in M1, ICMS at the recording sites of 414 neurons elicited movements of the digits, wrist, elbow, shoulder, or mouth (Table 1 and Fig 2). In Fig 3A–3E, we show typical examples of neurons, in which the timing of responses was consistent with the movements of the body part represented in the recording site. The neurons in the digit representation (*i*.*e*., the area in which ICMS at the recording sites elicited movements of the digits) showed peak activity in the period around AP or PO, when the digit movements were involved in grasping (Fig 3A and 3B). In contrast, the peak activity of the neurons in the wrist representation occurred before AP and around PO, with activity tending to decrease in between (Fig 3C), reflecting the fact that wrist movements mainly occurred during reaching and retrieval. The neuron in the elbow/shoulder representation continued to fire during the course of the task, and frequently showed peak firing during reaching and retrieval (Fig 3D). The neuron in the mouth representation showed peak activity just before or during HM when the monkey’s mouth started to open (Fig 3E).

**Table 1:** Number of primary motor cortex (M1) neurons analyzed.

	Digits	Wrist	Elbow/shoulder	Mouth
**Total**	140 (63, 77)	92 (52, 40)	79 (35, 44)	103 (63, 40)
**Movement thresholds ≤10 μA**	67 (25, 42)	41 (18, 23)	16 (7, 9)	45 (31, 14)
**Movement thresholds ≥15 μA**	73 (38, 35)	51 (34, 17)	63 (28, 35)	58 (32, 26)

The numbers for Monkeys B and G are shown in that order in parentheses.

We also observed neuronal responses that did not correspond to the body movements on the ICMS map, *e*.*g*., neurons in the digit representation that showed no statistically significant modulation during the task (Fig 3F), and those in the digit and mouth representations that showed peak activity in the periods around HM and AP, respectively (Fig 3G and 3H). The percentage of such neurons was higher in neurons located at recording sites with movement thresholds of at least 15 μA than in neurons located at recording sites with movement thresholds of 10 μA or less. For simplicity, hereafter we denote the former and latter as HT (higher threshold) neurons and LT (lower threshold) neurons, respectively. Note that the threshold is determined for the recording site and not for the actual neurons. Task-related changes in activity were not seen in 17.9% of HT neurons—a value significantly higher than that of LT neurons (3.0%; *P* < 0.01, chi-square test). Moreover, 30.2% of HT neurons in the mouth representation showed peak activity before 800 ms from HPR, when no mouth movement was observed. This value was significantly higher than that of LT neurons in the mouth representation (9.1%; *P* < 0.05, chi-square test).

### Population responses in recording sites with different movement thresholds

**Fig 4:**
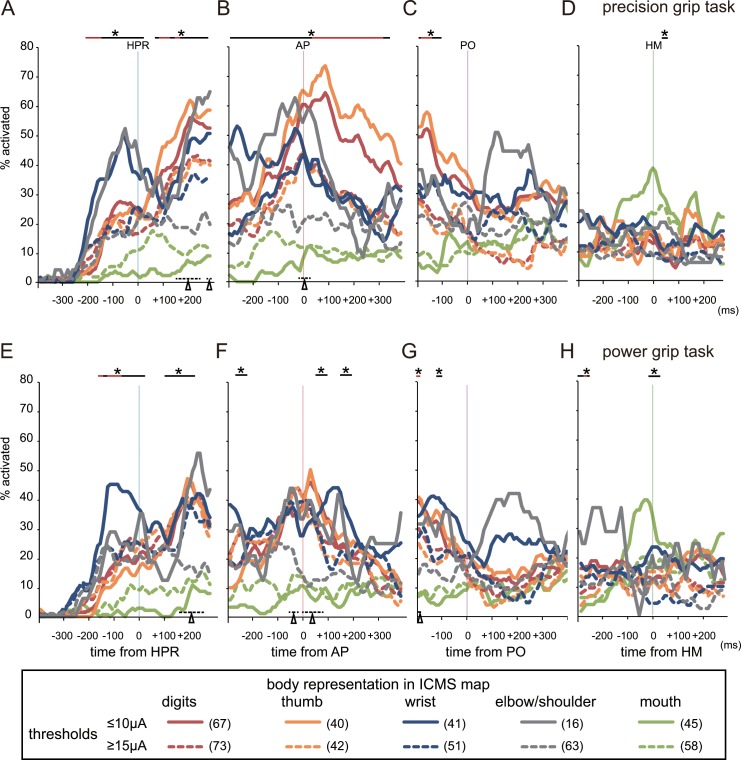
Percentage of neurons in each body representation of the intracortical microstimulation (ICMS) map in the primary motor cortex (M1) activated during the course of the task. (A–D) Precision grip task. (E–H) Power grip task. Neuronal firing rates were aligned to the time of homepad release (HPR: A, E), approaching the target (AP: B, F), pulling the target out (PO: C, G), and bringing the hand to the mouth (HM: D, H). Each line color represents a different body representation, and the solid and dashed lines show the results of lower threshold (LT) and higher threshold (HT) neurons located at recording sites with movement thresholds of 10 μA or less, and at least 15 μA, respectively. The number of neurons in each category is shown in parentheses. Chi-square tests were performed to test the null hypothesis that there is no association between the body representation and percentage of neurons activated, not only among the five body representations—digits, thumb, wrist, elbow/shoulder, and mouth—but also the four forelimb representations, excluding the mouth. The asterisks and solid lines at the top of the graphs indicate bins in which LT neurons in each of five representations were differentially activated (*P* < 0.01, chi-square test). The red lines indicate the bins in which LT neurons in each forelimb representation (i.e., without the mouth representation) were differentially activated (*P* < 0.01, chi-square test). Similarly, the black triangles and dashed lines at the bottom of the graphs indicate the 10-ms bins in which HT neurons in each representation including the mouth and each forelimb representation were differentially activated (*P* < 0.01, chi-square test). This analysis shows that populations of LT neurons in each body representation were differentially activated during reach-grasp-retrieval movements.

**Fig 5:**
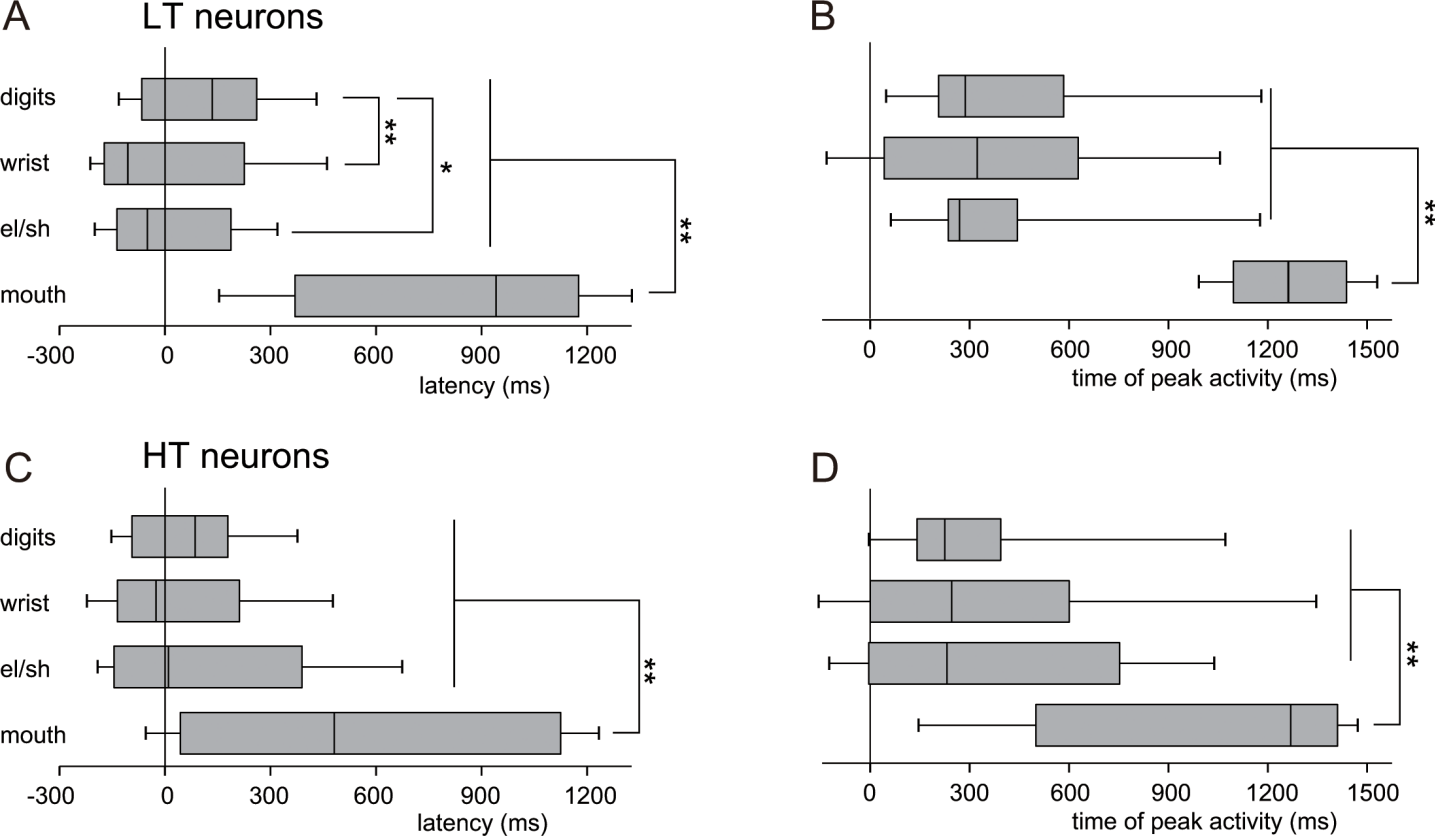
Sequential activation of neurons in each body representation of the ICMS map in M1 during task performance. (A, B) The box and whisker plots show the median, upper and lower quartiles, and 10th and 90th percentiles of the latencies (A) and the times of peak activity (B) relative to HPR of lower threshold (LT) neurons (located at recording sites with movement thresholds of 10 μA or less). Both latency and peak activation time of the neurons in the mouth representation were significantly greater than those in the forelimb representations, and the latency of the neurons in the digit representation was significantly greater than that in the wrist or elbow/shoulder representation (*P < 0.05, **P < 0.01, Mann–Whitney U-test). (C, D) The box and whisker plots show the median, upper and lower quartiles, and 10th and 90th percentiles of the latencies (C) as well as the times of peak activity (D) relative to HPR of higher threshold (HT) neurons (located at recording sites with movement thresholds of at least 15 μA). Both the latency and peak activation time of the neurons in the mouth representation were significantly greater than those in the forelimb representations (**P < 0.01, Mann–Whitney U-test). The black and white triangles indicate the median values of latency and time of peak activity for precision and power grip tasks, respectively.

To analyze the population responses of both LT and HT neurons, we investigated the percentage of neurons in each body representation of the ICMS map in M1 activated in each time bin during the course of the task (Fig 4). LT neurons in each representation—digits, thumb, wrist, elbow/shoulder, and mouth—were differentially activated during the precision grip task (solid lines and asterisks in Fig 4A–4D). The differential activity among LT neurons in each body representation during reaching (the period around HPR) reflects the fact that LT neurons in the wrist and elbow/shoulder representations were activated earlier than those in the digit representation (solid lines in Fig 4A). Moreover, the latency and time of peak activity of the neurons indicated that the latencies of LT neurons in the wrist and elbow/shoulder representations were significantly lower than those of LT neurons in the digit representation (*P* < 0.01 and *P* < 0.05, respectively, Mann–Whitney U-test, Fig 5A).

The differential activity among LT neurons in each body representation during grasping (the period between AP and PO) reflects the fact that more LT neurons in the digit representation were activated than those in the wrist and elbow/shoulder representations in the precision grip task (solid lines in Fig 4B and 4C). LT neurons in the mouth representation increased their activities at the period around HM (Fig 4D), and both the latency and time of peak activity of the neurons in the mouth representation were significantly greater than those in the forelimb representations (**P < 0.01, Mann–Whitney U-test, Fig 5A and 5B).

In contrast to LT neurons, there were few time periods when the population of HT neurons were differentially activated (dashed lines and triangles at the bottom of the graphs in Fig 4A–4D), and both the latency and time of peak activity of HT neurons were more variable than those of LT neurons (Fig 5C and 5D).

**Fig 6:**
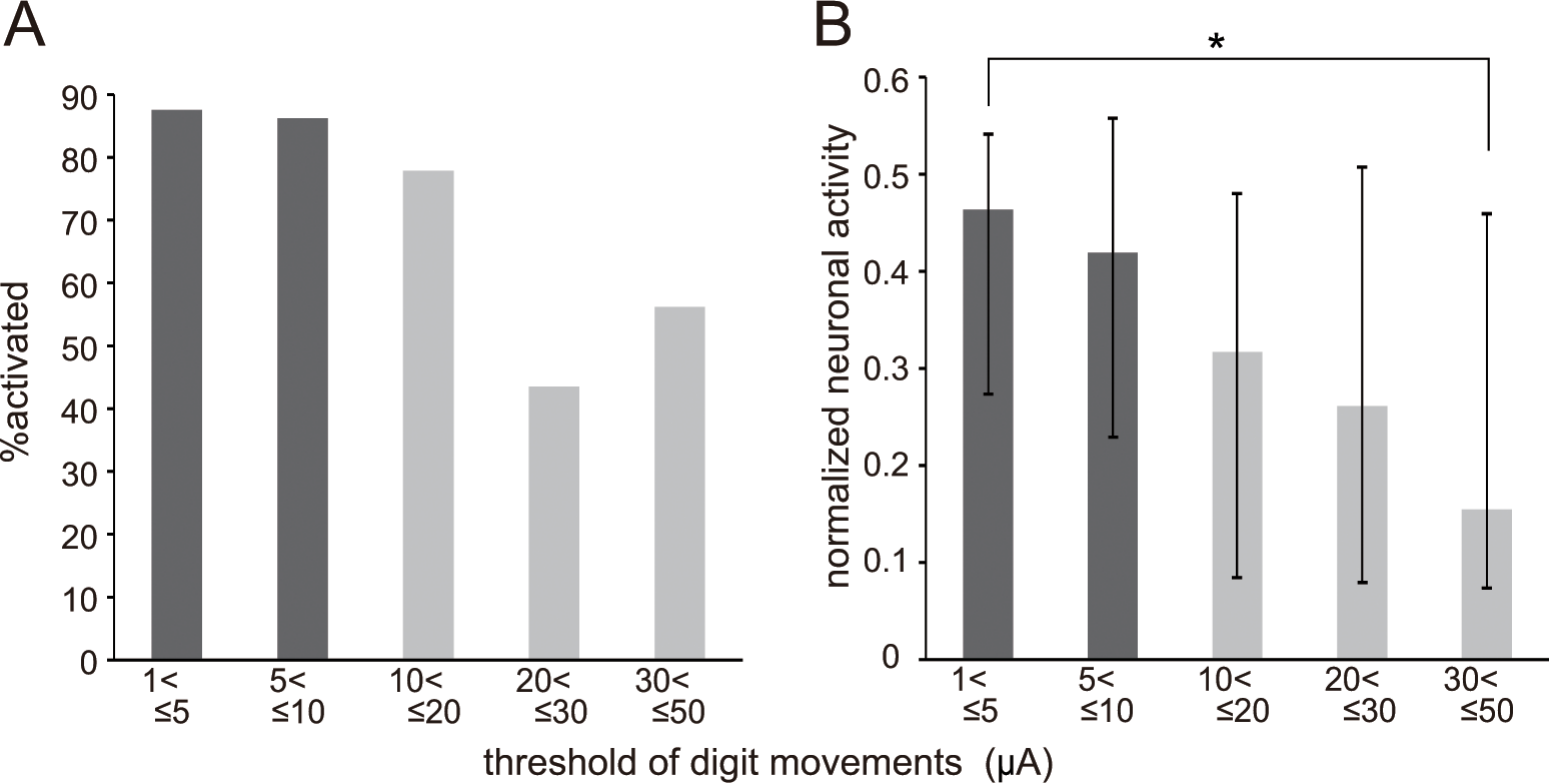
Difference of neural activity among neurons located at recording sites with different movement thresholds for intracortical microstimulation (ICMS). (A) Percentage of neurons in the digit representation of the ICMS map in the primary motor cortex (M1) activated during the grasping phase, 0 to +300 ms from the time of approaching the target (AP), of the precision grip task. (B) The median and interquartile range of normalized neuronal activities in the digit representation in the same period as (A). Both the percentage of activated neurons and normalized neuronal activity decreased as the movement threshold increased. The normalized neuronal activities for different movement thresholds were significantly different (*P* < 0.05, Kruskal–Wallis one-way ANOVA), and Dunn’s multiple comparisons test showed that the normalized neuronal activity for ≤5 μA was significantly greater than that for ≤50 μA (*P < 0.05). The numbers of neurons for ≤5 μA, ≤10 μA, ≤20 μA, ≤30 μA and ≤50 μA were 23, 40, 26, 22, and 22, respectively.

We arbitrarily divided the neurons into LT and HT neurons in the analyses described above; however, the movement thresholds of M1 varied from 1 to 50 μA. To further analyze how neuronal activity changes depending on the movement threshold, the neuronal response properties at recording sites of five different movement thresholds were compared (Fig 6). The percentage of activated neurons during the grasping phase (0 to +300 ms from AP) decreased sharply between neurons in the digit representation with movement thresholds of ≤20 μA and ≤30 μA (Fig 6A), whereas the normalized neuronal activity during the grasping phase monotonically decreased as the movement threshold increased (*P* < 0.05, Kruskal–Wallis one-way ANOVA, Fig 6B).

### Neuronal responses in different types of grasping

**Fig 7:**
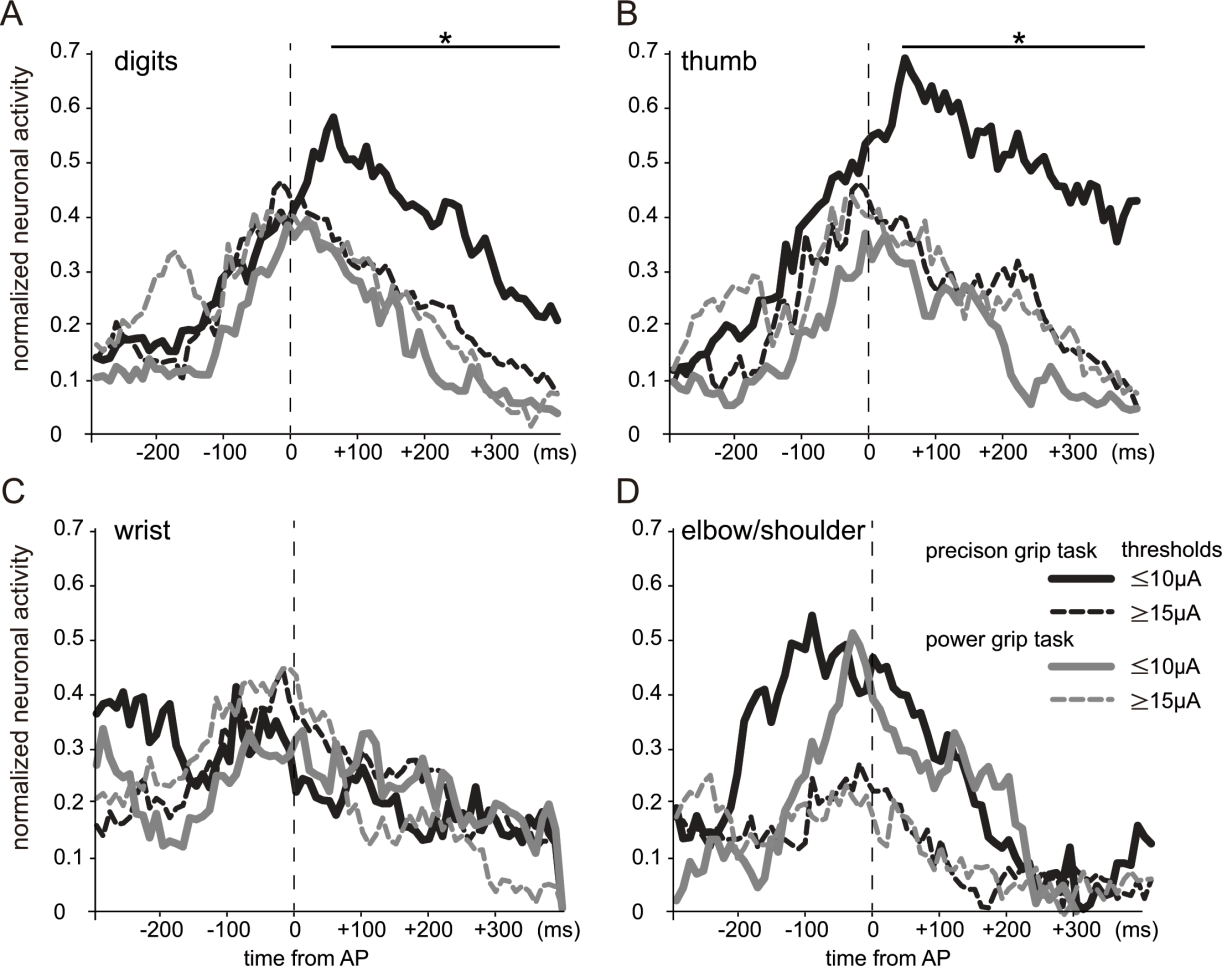
Normalized neuronal activity for both precision and power grip tasks, aligned to the time of approaching the target (AP), in each body representation in the primary motor cortex (M1). (A) Digit representation. (B) Thumb representation. (C) Wrist representation. (D) Elbow/shoulder representation. The neuronal firing rate was normalized by subtracting the baseline firing rate and then dividing the resulting value by the maximum level of activity observed across the two grasp types for all periods. The asterisks and lines at the top of the graphs indicate the bins in which the normalized activities of lower threshold (LT) neurons (located at recording sites with movement thresholds of 10 μA or less) for precision grasping were higher than those for power grasping (*P* < 0.001, Mann–Whitney U-test). In the digit representation, a significant difference in normalized neuronal activity was observed between precision and power gripping during the grasping phase, and the difference was greater when only neurons in the thumb representation were analyzed. LT neurons in the elbow/shoulder representation showed higher normalized neuronal activity than did HT neurons during the period just before grasping (–100 to 0 ms from AP) for both precision and power grip tasks (*P* < 0.01, Mann–Whitney U-test).

In the power grip task, activities of LT neurons during reaching (Fig 4E), retrieving (Fig 4G), and bringing the hand to the mouth (Fig 4H) were similar to those observed in the precision grip task (Fig 4A, 4C and 4D). However, the differences in activity among each body representation during grasping (the period between AP and PO) in the power grip task (Fig 4F) were less prominent, and the time periods with significantly different activation were shorter, than those during grasping in the precision grip task (Fig 4B). To clarify the difference in neural activity between precision gripping and power gripping, we calculated the normalized neuronal activity (Fig 7), which represents the relative magnitude of neuronal activity during both precision and power grip tasks (see *Materials and Methods* for details). In the digit representation, only the activity of LT neurons was significantly higher for the precision grip task than for the power grip task during the grasping phase (up to several hundred milliseconds from AP; black and gray solid lines and asterisks in Fig 7A); this difference was greater when only neurons in the thumb representation were analyzed (black and gray solid lines and asterisks in Fig 7B). In both the wrist and elbow/shoulder representations, no significant difference in normalized neuronal activity was observed between precision gripping and power gripping, even in LT neurons (Fig 7C and 7D). Interestingly, LT neurons in the elbow/shoulder representation (solid lines in Fig 7D) showed higher normalized neuronal activity than HT neurons (dashed lines in Fig 7D) during the period just before grasping (–100 to 0 ms from AP), for both precision and power grip tasks (*P* < 0.01, Mann–Whitney U-test), although no significant difference was observed between precision gripping and power gripping.

### Neuronal responses at different locations on the cortical surface

The median [interquartile range] depth of electrode penetration for LT neurons was 2270 [1390–3440] μm, whereas that for HT neurons was 2310 [850–4030] μm. The difference between them was not significant (*P* = 0.79, Mann–Whitney U-test). Although the depth does not always correspond to the cortical layers because a large portion of the macaque M1 is located within the central sulcus, the results suggest that the difference between LT and HT sites is not simply related to their distances from layer V but also to their locations on the cortical surface; that is, the difference may reflect the localization of neurons in different cortical columns.

**Fig 8:**
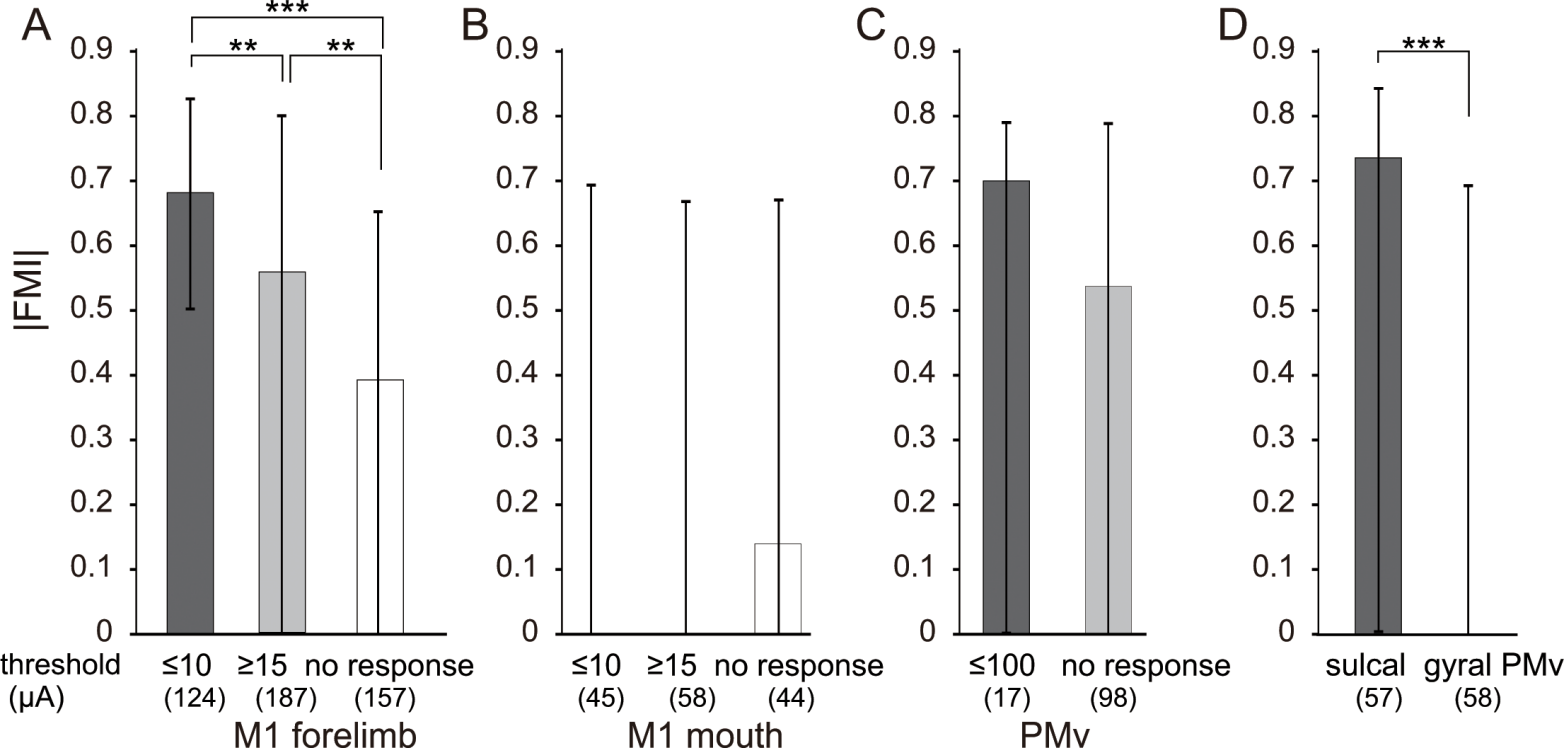
Variation of forelimb movement index (FMI) depending on both movement threshold for intracortical microstimulation (ICMS) and location on the cortical surface. (A) Median and interquartile range of |FMI| among recording sites at which ICMS evoked movements of the forelimb (i.e., digits, wrist, or elbow/shoulder) in the primary motor cortex (M1). The |FMI| values were compared across movement threshold ranges. ‘No response’ indicates recording sites of M1 at which ICMS up to 50 μA evoked no body movements, whereas ICMS within 2 mm of depth from the recording site in the same recording track evoked movements of the forelimb. The number of neurons in each category is shown in parentheses. Differences among the |FMI| values for different movement thresholds were evaluated using Kruskal–Wallis one-way ANOVA with Dunn’s test (**P < 0.01, ***P < 0.005). (B) Median and interquartile range of |FMI| in the mouth representation of M1. ‘No response’ is defined as in panel A. (C) Median and interquartile range of |FMI| in the ventral premotor cortex (PMv). Neurons in penetrations at which ICMS up to 100 μA evoked no body movements within PMv are denoted as ‘no response’. (D) Median and interquartile range of |FMI| are separately shown for the sulcal regions of PMv (within 2 mm of the arcuate sulcus) and the gyral regions of PMv. The index was higher in the sulcal regions than in the gyral regions of PMv (***P < 0.005, Mann–Whitney U-test). |FMI| is influenced by both baseline activity of neurons and modulation of activity during the task. In both M1 and PMv, the baseline activity was not significantly different among neurons with different movement thresholds (*P* > 0.3, Mann–Whitney U-test). In PMv, the baseline activities of the gyral regions were significantly higher than those in the sulcal regions (*P* < 0.01, Mann–Whitney U-test; the median values [interquartile range] in the sulcal and gyral PMv were 5.7 [2.9–10.4] and 8.6 [4.2–20.0]). Therefore, the difference in baseline activity between the sulcal and gyral regions in PMv accounts for roughly a 1.5-fold difference in |FMI| between the regions, and the difference in activity during task performance accounts for the rest of the difference in |FMI|.

We calculated the FMI, which represents how neural activity specifically changes during forelimb movements (see *Materials and Methods* for details). The percentage of neurons with negative FMI values, *i*.*e*., neurons with inhibited responses, was similar among areas and different movement thresholds. The percentages among HT and LT neurons were 8.1% and 12.4%, respectively, in the forelimb representation and 11.1% and 10.3% in the mouth representation. The percentages of neurons with negative FMI among penetrations in PMv where ICMS did or did not evoke forelimb movements were 11.8 and 9.6%, respectively. No statistical difference was observed among them (*P* > 0.3, chi-square test). In Fig 8, we present the absolute value of the FMI, *i*.*e*., |FMI|, to show the extent to which each category of neurons was modulated during forelimb movements. This analysis indicates that the |FMI| for different movement thresholds was significantly different in the forelimb representations of the ICMS map in M1 (P < 0.01, Kruskal–Wallis one-way ANOVA with Dunn’s test, Fig 8A). The median |FMI| value was zero at recording sites where ICMS at ≤10μA evoked mouth movements, whereas a certain percentage of neurons in ‘no response’ recording sites showed an |FMI| above zero (Fig 8B).

A number of neurons in PMv showed changes in firing during task phases in which movements of the forelimb such as reaching, grasping, or retrieving occurred (Fig 3I). In contrast to the results in M1, in PMv we did not observe a correlation between the response properties of neurons as indicated by |FMI| and the body representation in the ICMS map. Although the median |FMI| was slightly higher at penetrations where ICMS up to 100 μA evoked forelimb movements than at those where it evoked no movement, the difference was not significant (Fig 8C). Neural responses specific to forelimb movements were often observed in the rostral part of PMv including the lateral bank of the lower arcuate limb, in which ICMS up to 100 μA evoked no detectable movement. The median |FMI| in the sulcal regions of PMv, within 2 mm of the arcuate sulcus, was significantly higher than that in the gyral regions of PMv (*P* < 0.005, Mann–Whitney U-test, Fig 8D).

## Discussion

### Difference of neuronal responses depending on the movement threshold of ICMS

The present study showed that the population responses of LT neurons in each body representation of M1 were different during the reach-grasp-retrieval task, and the timing of neuronal responses was consistent with the movements of the body part represented. Moreover, neurons in the digit representation responded differently for the different types of grasping. The results suggest that the topographic body map in M1 derived by ICMS with conventional stimulation parameters generally corresponds to functional divisions of voluntary movements, supporting the conventional view that the motor cortex contains a body map used to control movements of each body part. As described in the *Introduction*, ICMS with conventional stimulation parameters was found to contain several issues (*e*.*g*., trans-synaptic excitation). Nevertheless, conventional ICMS may still be useful for identifying functional divisions in M1. In addition, the present study showed that neural activity depends on the ICMS current threshold required to elicit body movements and the location on the cortical surface.

In contrast to LT neurons, some responses of HT neurons did not correspond to body movements; the latency and time of peak activity of HT neurons showed more variability and little difference among the body representations. These results raise the possibility that body movements produced by higher electrical current may not always be physiologically relevant. Based on previous estimates of the effective radius of conventional ICMS to excite neuronal cell bodies and axons [4, 37–39], the effective radius at 10 μA ranges from 50 to 300 μm. Physiological studies have reported that the activities of motor cortex neurons within approximately 200 or 240 μm are summed to represent a kinematic parameter of movement [40, 41], and an anatomical study has shown that the somata and dendritic trees of M1 neurons are grouped into columnar aggregations of 100 to 300 μm in width [42]. These studies have suggested a width of 100–300 μm for the functional cortical columns in M1 [43], and the involvement of these columns in body movements was indicated by a recent fiber-optic calcium recording study [29]. Thus, conventional ICMS using an electrical current lower than 10 μA, whose effective radius is up to 300 μm, is considered appropriate for activating neurons within a single functional cortical column in M1.

Our finding that LT neurons in the digit representation were activated more during precision gripping than during power gripping is consistent with the results of previous studies showing that, compared with power gripping, precision gripping is associated with higher firing rates of pyramidal tract neurons [44–46]. The differential activity may reflect the need for finer digit control in precision gripping than in power gripping. Interestingly, LT neurons in the elbow/shoulder representation showed higher normalized neuronal activity than did HT neurons during the period just before grasping for both precision and power grip tasks. Grasping depends on correct positioning of the hand and wrist, and therefore fine and coordinated control of the proximal joints, including the elbow and shoulder, is important to perform reach-to-grasp movements [47–51]. The present result suggests that LT neurons in the elbow/shoulder representation, which probably include neurons whose axons extend into the corticospinal tracts [52, 53], may be involved in the fine control of shoulder movements during grasping.

### Difference of neuronal responses within and between areas

The result in PMv, which showed no correlation between the response properties of neurons and the body representation in the ICMS map, is consistent with a previous study reporting that modulation of neural activity associated with reaching or grasping did not correlate with the movements evoked by ICMS in the recording site within PMv [35]. The present study also investigated the spatial distribution of neural activity related to forelimb movements, including an area of PMv where ICMS evoked no movement, and showed that higher forelimb movement–related activity was observed in the recording sites around the arcuate sulcus. Our recent brain imaging study showed that activity of the sulcal PMv increased during the post-recovery phase after an irreversible lesion of the M1 hand area [13]. Pharmacological inactivation by muscimol indicated that the increased activity observed in the sulcal PMv was involved in functional recovery from the motor deficit caused by the M1 lesion [13]. Taken together, the sulcal PMv may be more directly involved in forelimb movements than the gyral region of PMv, and may have the potential to take over functions of the M1 hand area in the event that it is damaged.

An important next step will be to investigate how neuronal activities in each body representation in both M1 and PMv change during the post-recovery phase after an irreversible lesion of the M1 hand area. The present study provides essential baseline data for future research on the topic.

## Supporting Information

S1 ARRIVE Guidelines Checklist We followed the ARRIVE (Animal Research: Reporting of *In Vivo* Experiments) guidelines and the ARRIVE Checklist is available. (PDF)
